# Satisfaction levels and associated influencing factors among inpatients and outpatients with breast cancer at a tertiary health facility in Ghana

**DOI:** 10.4314/gmj.v58i1.3

**Published:** 2024-03

**Authors:** Benedict NL Calys-Tagoe, Kirstyn Brownson, Josephine Nsaful, Florence Dedey, John Tetteh, Nathaniel Coleman, Ruth Y Laryea, Joe-Nat Clegg-Lamptey

**Affiliations:** 1 Department of Community Health, University of Ghana Medical School, Korle Bu, Accra; 2 Huntsman Cancer Institute, University of Utah School of Medicine, Utah, USA; 3 Department of Surgery, University of Ghana Medical School, College of Health Sciences, Korle Bu, Accra; 4 Department of Surgery, Korle Bu Teaching Hospital, Accra, Ghana; 5 Department of Obstetrics and Gynaecology, University of Ghana Medical School, Korle Bu, Accra; 6 Department of Medicine and Therapeutics, University of Ghana Medical School, Korle Bu, Accra

**Keywords:** Breast cancer, satisfaction level, KBTH, inpatients, outpatients, Ghana

## Abstract

**Objectives:**

To uncover variables linked to breast cancer patient satisfaction in order to improve policy choices and actions for breast cancer care in Ghana.

**Design:**

We employed a cross-sectional design using a quantitative approach

**Setting:**

The Radiotherapy, Oncology and Surgery Departments of the Korle Bu Teaching Hospital, Accra

**Participants:**

Inpatient and outpatient breast cancer patients.

**Main outcome measures:**

The level of inpatient and outpatient satisfaction was measured using descriptive and inferential statistical analyses. The Shapiro-Wilk test was employed to assess normality, while the Heckman selection model assessed significance with outcomes of interest.

**Results:**

A total of 636 participants, with a mean age of 52.64±14.07 years, were recruited. The measured inpatient and outpatient levels of satisfaction out of 100 were 74.06±7.41 and 49.99±1.00 respectively, while the self-reported satisfaction levels out of 5 were 4.22±0.63 and 4.11±0.85 respectively. The level of inpatient satisfaction was significantly influenced by age, marital status, income level, and number of previous facilities visited (p<0.05). Outpatient satisfaction level was significantly associated with place of residence and income level (p<0.05).

**Conclusions:**

The study offers insight into the satisfaction levels of breast cancer patients receiving inpatient and outpatient services at the largest tertiary referral centre and teaching hospital in Ghana, as well as the factors influencing attendance and satisfaction levels. Understanding and improving breast cancer patients' levels of satisfaction is a way that providers can safeguard their emotional well-being. Improvement in patient satisfaction at our institution among outpatients is an area for future growth.

**Funding:**

Gardner-Holt Women's Health Grant program, Centre for Global Surgery 2021

## Introduction

Breast cancer is a serious public health issue with more than 2 million women diagnosed globally each year.^1^,^2^ Although there is no population-based national cancer registry in Ghana, WHO Globocan 2020 estimates that breast cancer accounted for 24.9% of all cancers and 31.8% of female cancers in Ghana that year. 2 The Korle Bu Cancer Registry, located in the largest referral hospital in the country, reported breast cancer incidence as 40.8% of all female cancers in 2014.^3^ About 70% of women newly diagnosed with breast cancer in Ghana present with advanced breast disease. 4 Breast cancer diagnosis and treatment directly affects women physically, psychologically, and socially and negatively impacts their family and social relationships.^5^ Bolstering breast cancer patient satisfaction in the inpatient and outpatient setting is a way for breast cancer care providers to support their patients' emotional wellbeing. In the competitive world of healthcare the value of patient satisfaction and quality of life is increasingly being taken into consideration.^6^

Patient satisfaction has received a lot of attention, and the health system has made it one of its key goals. Any system that wishes to become more effective must be conscious of and take into account the opinions of its users.^7^ Access to patients' opinions is an important approach to assessing the quality of health care services and thus it is important to understand patient expectations.^7^,^8^ Patients are important sources of information for evaluating the quality of care because they are the hospital's main clientele, and their contentment can in some cases indicate that services are being provided properly when their satisfaction is being achieved. 9

Patient satisfaction is a cognitive response that is influenced by various factors such as accessibility and continuity of medical and paramedical care, financial costs, interpersonal aspects such as reception and orientation, and facility interactions between patients and caregivers.^8^,^10^-^12^ The level of patient satisfaction reflects the quality of care provided by facilities based on these factors. In this regard, assessing patient satisfaction by identifying factors that influence satisfaction, comprehending patient needs and expectations, and removing dissatisfying factors can improve service delivery^10^ resulting in increased patient satisfaction and improved physical and mental health.^12^ Health managers and policymakers can benefit from the evaluation of this index by learning more about the quality and quantity of process improvement activities as well as quality improvement.^13^ The implementation of a strategy for providing patients with the best care is made possible by measuring this level of satisfaction and the factors affecting it.^11^ Studying patient satisfaction and patient experience can help improve patient satisfaction and experience for future patients, thus helping breast cancer patients who are struggling with the physical, social, and psychological effects of their cancer treatments.

This current study focused on patient satisfaction amongst both inpatient and outpatient breast cancer patients treated at the largest referral center and teaching hospital in Ghana. The study aims to assess levels of patient satisfaction and uncover variables linked to breast cancer patient satisfaction in order to shape future interventions that can improve patient care.

## Methods

### Study design

This study employed a cross sectional design using a quantitative approach to assess satisfaction with care received at Korle Bu Teaching Hospital (KBTH) by breast cancer patients in both the inpatient and outpatient setting.

### Study site

KBTH is the largest referral center and teaching hospital in Ghana with over 2000 beds. It has a dedicated surgical breast unit, an oncology and radiotherapy center, and offers services for breast cancer diagnosis, staging, treatment, and palliative care. Treatments offered at KBTH include surgery, radiation, chemotherapy, endocrine therapy, and immunotherapy. The hospital has an active multidisciplinary team for breast cancer management.

### Study participants

Participants for this study were breast cancer patients receiving care at KBTH. Females with a histologic diagnosis of breast cancer who have been receiving breast cancer treatment at the Surgical Department and/or the National Centre for Radiotherapy, Oncology and Nuclear Medicine at KBTH for at least three months and who consented to be part of the study were included. Male breast cancer patients were excluded.

### Sample size and sampling technique

Data was collected over a one-year period from January 2022 through December 2022. Consecutive breast cancer patients receiving care at KBTH during the data collection period who met the inclusion criteria were recruited to participate in the study. Participants were selected from several sites including the surgical Outpatient Department (OPD), chemotherapy suite, surgical wards, and the radiotherapy OPD. A total of 636 participants participated in this study. Measures were put in place to avoid multiple recruitment of the same individual.

### Procedures

A structured, interviewer-administered questionnaire was used for data collection. This involved gathering data regarding patient satisfaction with the care they had received at the hospital. Additionally, patients were surveyed to assess factors that may have caused delays in their presentation to the hospital for treatment. The interviews were conducted in person on the hospital premises after recruitment by trained research assistants who were not part of the medical team taking care of the patient. Inpatients were asked; “*Have you been admitted for breast cancer surgery?”* Outpatients were asked, “*Have you been attended to at the surgical or* National Centre for Radiotherapy, Oncology and Nuclear Medicine *Outpatient Department (OPD)?*” If the response to either question was “yes,” the applicable satisfaction questions were further asked.

### Outcome measurement

The two primary outcomes that were assessed among breast cancer patients treated at KBTH were a) level of patient satisfaction with inpatient care and b) level of patient satisfaction with outpatient care. Satisfaction variable was measured among inpatients and outpatients inclusively.

An 18-point Likert Scale questionnaire (Table 1) was administered to inpatients and a 23-point Likert Scale questionnaire (Table 2) was administered to outpatient participants. The overall raw scores were normalized to standardize the values into t-scores of normality. After normalization, the values were normally distributed using the Shapiro-Wilk test of normality. Assessment of patient satisfaction was a selective estimate because it emanated from being either attended to as an inpatient and/or outpatient separately. Self-reported satisfaction was assessed by patients' response to the question, “Overall, how satisfied are you with the level of care received?” The responses ranged from 1-5, with 1 being the least satisfied and 5 denoting most satisfied. The reliability score for inpatient and outpatient satisfaction was approximately 80% and 88% respectively (Tables 2 and 3). Data analysis was presented in two approaches: descriptive and inferential. The descriptive analysis was reported in the form of frequencies, percentages, and where applicable the mean and standard deviation were also presented. The Shapiro-Wilk test of normality was employed to assess normal distribution of continuous variables. The proportion of inpatient and outpatient attendance rate was estimated by the demographic characteristics. Significant differences in the proportions were analyzed using a test of non-linear estimation parameter post estimation.

**Table 1:** Socio-demographic characteristics and prevalence of inpatient and outpatient attendance among breast cancer patients

Variable	Frequency	Inpatients (392)	Outpatients (475)
	n(%)	% [95% CI]	% [95% CI]
**Overall**	N=636	61.63[57.78 to 65.34]	74.68[71.16 to 77.92]
**Age (years)**			
**≤39**	97(15.25)	57.73[47.71 to 67.16]	74.23[64.61 to 81.96]
**40-49**	171(26.89)	56.73[49.19 to 63.96]	75.44[68.42 to 81.32]
**50-59**	187(29.4)	62.03[54.86 to 68.71]	73.26[66.45 to 79.12]
**60+**	181(28.46)	67.96[60.80 to 74.36]	75.69[68.90 to 81.40]
**Mean±SD**	52.64±12.07		
**Test**		5.61	0.36
**Religion**			
**Traditional**	7(1.10)	85.71[41.84 to 98.04]	85.62[41.84 to 98.10]
**Christianity**	579(91.04)	61.14[57.09 to 65.04]	74.96[71.26 to 78.32]
**Islam**	50(7.86)	64.00[49.92 to 76.02]	70.00[56.00 to 81.05]
**Test**		3.48	1.24
**Level of education**			
**No formal education**	53(8.33)	73.58[60.17 to 83.70]	81.13[68.33 to 89.55]
**Primary**	55(8.65)	60.00[46.63 to 72.03]	69.09[55.76 to 79.86]
**Middle/ Junior High School**	162(25.47)	58.64[50.90 to 65.98]	75.31[68.08 to 81.35]
**Secondary**	163(25.63)	63.80[56.14 to 70.83]	76.69[69.57 to 82.56]
**Tertiary**	203(31.92)	59.61[52.70 to 66.15]	72.41[65.85 to 78.13]
**Test**		5.21	3.15
**Marital status**			
**Never married**	77(12.11)	62.34[51.05 to 72.43]	76.62[65.89 to 84.76]
**Married**	358(56.29)	57.26[52.07 to 62.30]	69.55[64.58 to 74.11]
**Co-habiting**	7(1.1)	85.71[41.84 to 98.04]	85.71[41.84 to 98.04]
**Divorced/Separated**	98(15.41)	67.35[57.47 to 75.89]	82.65[73.83 to 88.94]
**Widowed**	96(15.09)	69.79[59.89 to 78.14]	83.33[74.49 to 89.54]
**Test**		10.53*	14.32**
**Place of residence**			
**Urban**	560(88.05)	62.67[58.58 to 66.60]	76.25[72.54 to 79.60]
**Peri-urban**	60(9.43)	58.33[45.57 to 70.07]	65.00[52.19 to 75.96]
**Rural**	16(2.52)	37.50[17.87 to 62.32]	56.25[32.34 to 77.57]
**Test**		4.51	5.41
**Employment status**			
**Not employed**	198(31.13)	63.64[56.70 to 70.05]	74.75[68.22 to 80.32]
**Employed**	438(68.87)	60.73[56.07 to 65.21]	74.66[70.36 to 78.52]
**Test**		0.49	0.01
**Income (GHS)**			
**<500**	176(27.89)	61.36[53.96 to 68.28]	79.55[72.93 to 84.88]
**500-1000**	85(13.47)	55.29[44.62 to 65.50]	68.24[57.62 to 77.24]
**1001-2000**	92(14.58)	60.87[50.56 to 70.29]	69.57[59.43 to 78.10]
**2001-5000**	40(6.34)	70.00[54.23 to 82.13]	77.50[62.09 to 87.87]
**>5000**	238(37.72)	63.45[57.13 to 69.33]	74.37[68.43 to 79.52]
**Test**		3.06	5.5
**First point of call**			
**Health facility**	576(90.57)	60.76[56.71 to 64.68]	73.44[69.67 to 76.89]
**Other**	60(9.43)	70.00[57.30 to 80.23]	86.67[75.51 to 93.20]
**Test**		2.18	7.73**
**Previous number of health facilities visited**			
**None**	61(9.59)	80.33[68.45 to 88.49]	91.80[81.75 to 96.55]
**1 facility**	331(52.04)	64.35[59.03 to 69.34]	77.95[73.15 to 82.10]
**2 facilities**	188(29.56)	52.66[45.50 to 59.71]	65.96[58.88 to 72.39]
**3+ facilities**	56(8.81)	55.36[42.24 to 67.76]	66.07[52.80 to 77.22]
**Test**		21.21***	31.04***
**Hospital visit was influenced by another**			
**No**	411(64.62)	59.85[55.03 to 64.50]	73.97[69.50 to 77.99]
**Yes**	225(35.38)	64.89[58.42 to 70.86]	76.00[69.98 to 81.14]
**Test**		1.59	0.32
**Hospital visit was based on personal conviction**			
**No**	324(50.94)	66.67[61.34 to 71.59]	76.23[71.29 to 80.56]
**Yes**	312(49.06)	56.41[50.84 to 61.82]	73.08[67.87 to 77.72]
**Test**		714***	0.84

NOTE: Unpaired t-test was employed to assess significant mean difference between the outcomes. Abbreviation: CI=Confidence Interval; SD=Standard Deviation; Min=Minimum; Max=Maximum; GHS=Ghana Cedis. P-value notation

* p<0.05

** p<0.01

*** p<0.001

**Table 2:** Inpatient satisfaction test of reliability scores

Item	n	Sign	Item-test correlation	Item-rest correlation	Average interitem covariance	alpha
**The nature of the surgery and complications were adequately explained**	392	+	0.32	0.22	0.14	0.79
**My concerns were addressed and questions answered**	392	+	0.49	0.42	0.14	0.78
**I was admitted promptly**	392	+	0.51	0.42	0.13	0.78
**Admitting nurses treated me with respect and courtesy**	392	+	0.60	0.55	0.13	0.78
**Nurses treated me with respect and dignity throughout my stay**	392	+	0.62	0.55	0.13	0.78
**Doctors treated me with respect and courtesy throughout my stay**	392	+	0.64	0.60	0.14	0.78
**Other staff treated me with respect and courtesy throughout my stay**	392	+	0.56	0.50	0.13	0.78
**My privacy was respected**	392	+	0.62	0.55	0.13	0.78
**Breast care nurses, counsellors are supportive whilst on admission**	392	+	0.35	0.22	0.14	0.80
**If I had pain, staff attended to me promptly**	392	+	0.53	0.43	0.13	0.78
**I was provided with clear follow-up and discharge instructions**	392	+	0.58	0.51	0.13	0.78
**After discharge wound care and dressings were done regularly and promptly**	392	+	0.57	0.50	0.13	0.78
**The cleanliness of the ward was adequate**	392	+	0.61	0.53	0.13	0.78
**The cleanliness of the bathroom was adequate**	392	+	0.58	0.47	0.13	0.78
**The quality of the food served was reasonable**	392	+	0.31	0.18	0.14	0.80
**I was given an estimate of the hospital bill before admission**	392	+	0.27	0.09	0.15	0.82
**Hospital bill was reasonable**	392	+	0.38	0.23	0.14	0.80
**Overall, I am satisfied with the inpatient services**	392	+	0.66	0.60	0.13	0.78
**Test scale**					0.13	0.80

**Table 3:** Outpatient satisfaction test of reliability scores

Item	n	Sign	Item-test correlation	Item-rest correlation	Average inter-item covariance	Alpha
***General Services***						
**How long you have to wait to be seen by a doctor**	475	+	0.37	0.27	0.19	0.89
**The privacy you get at the consultation room**	475	+	0.46	0.40	0.19	0.88
**How well you understand the information and instructions given you**	475	+	0.46	0.39	0.19	0.88
**The cleanliness of the waiting area, toilets, etc**	475	+	0.26	0.15	0.19	0.89
**The cost of services at the OPD**	475	+	0.36	0.27	0.19	0.88
**How OPD office staff treat you**	475	+	0.46	0.38	0.19	0.88
**How OPD nurses treat you**	475	+	0.54	0.48	0.18	0.88
***Doctors***						
**Easy to talk to**	475	+	0.50	0.45	0.19	0.88
**Adequately explained diagnosis and treatment plan**	475	+	0.55	0.49	0.19	0.88
**Answered all your questions**	475	+	0.57	0.53	0.18	0.88
**Involved you in decision making**	475	+	0.53	0.48	0.18	0.88
**Reassuring, made you comfortable**	475	+	0.60	0.55	0.18	0.88
**Understood you, sensitive**	475	+	0.57	0.52	0.19	0.88
**Available when you had concerns**	475	+	0.56	0.51	0.18	0.88
***Breast Care Nurses, Counsellors***						
**Easy to talk to**	475	+	0.63	0.58	0.18	0.87
**Answered all your questions**	475	+	0.67	0.62	0.18	0.87
**Reassuring, made you comfortable**	475	+	0.64	0.59	0.18	0.87
**Understood you, sensitive**	475	+	0.67	0.62	0.18	0.87
**Available when you had concerns**	475	+	0.70	0.66	0.18	0.87
**Helpful with directions, instructions, appointments, etc**	475	+	0.68	0.63	0.18	0.87
**Able to contact them by phone**	475	+	0.53	0.45	0.18	0.88
**They sometimes contact you by phone**	475	+	0.54	0.46	0.18	0.88
**Overall, I am satisfied with outpatient services**	475	+	0.71	0.67	0.18	0.87
**Test scale**					0.18	0.88

Inferential analysis involving significant mean difference and associations was performed. Significant mean difference employed t-test or Analysis of Variance depending on the category of the response variable. The Heckman selection model was adopted to assess significant factors associated with the outcomes of interest. In this current study, the measured satisfaction level for inpatient and outpatient participants was assessed inclusively. The overall scores were normalized into percentages ranging from 0-100%.

The Heckman selection model was applied to control for selection bias for the satisfaction outcomes. This technique is appropriate to produce unbiased estimates based on selectivity (i.e inpatient or outpatient attendance coded as 1=yes and 0=no) even when missing data are systematically related to unobserved characteristics.^14^

### Ethical considerations

The study protocol was reviewed and approved by the Korle Bu Teaching Hospital Institutional Review Board (KBTH-STC/IRB/00099/2021). Written informed consent was obtained from all participants and research was conducted in line with standard research ethical principles. To ensure confidentiality, no participant identifying details (names) were used in any form or in reports.

## Results

The study involved a total of 636 breast cancer patients visiting the KBTH for clinical services. Their ages ranged from 19-86 years with the mean age ± SD being 52.64±14.07 years. Most patients identified as being of Christian faith (91.04%) and a little less than one-third had tertiary level education (31.92%). More than half were married (56.29%) and approximately 88% resided in an urban setting (Table 3). Three quarters of patients (74.68%) (95% CI=71.16 to 77.92) had experiences as outpatients while 61.63% (95% CI=57.78 to 65.34) had experiences as inpatients. The differences in the proportion of inpatients with respect to the independent variables was found to be significantly associated with the following variables: “marital status,” “previous number of health facilities visited,” and “reason for visiting the facility,” while the differences in the proportion of outpatients with respect to the independent variables was found to be significantly associated with: “marital status,” “first point of call,” and “previous number of health facilities visited” (p<0.05) (Table 1).

The measured inpatient and outpatient levels of satisfaction on a scale of 0-100 were 74.06±7.41 and 49.99±1.00 respectively. Meanwhile, the self-reported satisfaction levels on a scale of 0-5 were 4.22±0.63 and 4.11±0.85 respectively. Though the measured satisfaction level for inpatients was much higher than that for outpatients, from unpaired t-test analysis, the differences in these estimates were not statistically significant (p>0.05). (see Tables 2 and 3). For inpatients, level of satisfaction by independent variable was significantly associated with participant reason for visiting the health facility. Those who visited the health facility because of their personal conviction about the capability of the facility in helping solve their problem had significantly higher satisfaction levels compared to their counterparts without such personal conviction (75.31±7.52 versus 73.04±7.17 respectively).

For outpatients, differences in satisfaction levels were significantly associated with place of residence (p<0.05). Those living in rural areas had higher satisfaction levels than those from urban or peri-urban areas (51.07±0.67) (Table 4).

**Table 4:** Level of satisfaction among breast cancer patients who received inpatient and outpatient clinical services

Variable	Inpatient (392)		Outpatient (475)	
	Measured	Self-reported	Measured	Self-reported
	Mean±SD	Mean±SD	Mean±SD	Mean±SD
**Overall**	74.06±7.41	4.22±0.63	49.99±1.00	4.11±0.85
**Age**				
**≤39**	75.18±7.24	4.25±0.61	49.86±0.93	4.03±0.87
**40-49**	74.47±8.21	4.23±0.82	50.11±1.10	4.18±0.90
**50-59**	73.81±6.97	4.24±0.50	50.02±0.95	4.15±0.76
**60+**	73.46±7.23	4.19±0.58	49.95±0.98	4.05±0.89
**Test**	0.84	0.2	1.17	0.84
**Religion**				
**No religion**	75.00±9.49	4.25±0.50	50.68±0.70	4.25±0.96
**Christianity**	74.08±7.46	4.24±0.60	49.98±0.99	4.10±0.85
**Islam**	79.00±1.41	4.00±0.88	50.11±1.18	4.26±0.85
**Test**	0.39	1.56	0.85	0.74
**Level of education**				
**No formal education**	73.23±7.20	4.03±0.78	49.98±0.97	4.12±0.73
**Primary**	71.33±7.05	4.12±0.60	50.04±1.10	3.97±0.85
**Middle/ Junior High School**	75.04±7.05	4.25±0.68	50.00±0.92	4.20±0.79
**Secondary**	73.92±7.72	4.24±0.55	50.01±0.98	4.14±0.89
**Tertiary**	74.42±7.48	4.27±0.61	49.99±1.07	4.04±0.90
**Test**	1.75	1.43	0.02	0.92
**Marital status**				
**Never married**	73.85±8.05	4.23±0.81	49.74±1.18	3.81±1.01
**Married**	74.10±7.18	4.21±0.59	50.06±0.97	4.18±0.83
**Co-habiting**	71.17±11.25	4.00±0.63	49.65±0.85	4.00±0.89
**Divorced/Separated**	73.94±7.60	4.18±0.63	49.95±0.99	4.04±0.83
**Widowed**	74.48±7.23	4.31±0.63	50.09±0.96	4.20±0.75
**Test**	0.29	0.62	1.61	2.65
**Place of residence**				
**Urban**	73.86±7.47	4.21±0.64	49.99±1.01	4.10±0.85
**Peri-urban**	75.74±6.57	4.31±0.53	49.84±0.85	4.05±0.86
**Rural**	76.00±7.92	4.33±0.52	51.07±0.67	4.89±0.33
**Test**	1.24	1.56	5.72***	0.74
**Employment status**				
**Not employed**	73.54±7.72	4.16±0.69	49.98±1.05	4.09±0.88
**Employed**	74.31±7.26	4.25±0.60	50.04±0.98	4.12±0.84
**Test**	-0.96	-1.37	-0.28	-0.41
**Income (GHS)**				
**<500**	74.53±7.26	4.24±0.62	50.13±0.96	4.20±0.80
**500-1000**	74.04±7.78	4.23±0.62	49.88±1.02	4.13±0.86
**1001-2000**	73.06±8.19	4.23±0.67	50.13±0.98	4.21±0.81
**2001-5000**	73.98±6.90	4.25±0.55	49.72±1.05	3.92±0.88
**>5000**	73.86±6.31	4.11±0.74	50.31±0.86	3.87±1.06
**Test**	0.76	1.56	0.85	0.74
**First point of call**				
**Health facility**	74.26±7.28	4.23±0.63	50.01±1.01	4.12±0.86
**Other**	72.40±8.25	4.19±0.63	49.93±0.95	4.02±0.80
**Test**	1.54	0.34	0.52	0.83
**Previous number of health facilities visited**				
**None**	74.65±6.76	4.27±0.67	50.11±0.88	4.16±0.78
**1 facility**	73.72±7.53	4.22±0.60	49.91±1.04	4.09±0.84
**2 facilities**	74.77±7.09	4.19±0.70	50.12±0.94	4.15±0.92
**3+ facilities**	73.23±8.58	4.26±0.58	50.04±1.07	4.08±0.80
**Test**	0.39	1.56	0.85	0.74
Hospital visit was influenced by another				
**No**	74.38±7.61	4.26±0.69	49.97±1.03	4.11±0.87
**Yes**	73.52±7.04	4.16±0.51	50.05±0.95	4.11±0.82
**Test**	1.11	1.56	-0.74	0.01
Hospital visit was based on personal conviction				
**No**	73.04±7.17	4.12±0.60	50.02±0.99	4.06±0.81
**Yes**	75.31±7.52	4.35±0.65	49.97±1.01	4.17±0.89
**Test**	-3.05**	-3.58***	1.72	-1.47

NOTE: Unpaired t-test was employed to assess significant mean difference between the outcomes. Differences between independent variable were assessed using the t-test or ANOVA depending on the category of the response variable. Abbreviation: CI=Confidence Interval; SD=Standard Deviation; GHS=Ghana Cedis. P-value notation:

** p<0.01

*** p<0.001

After applying the Heckman selection model to control for selection bias for the satisfaction outcomes, level of inpatient satisfaction was significantly influenced by age, marital status, and number of previous facilities visited (p<0.05). Increasing age significantly increased inpatient satisfaction by 0.01% (95% CI=0.00 to 0.02). Co-habitation significantly increased inpatient satisfaction by 5.93% (95% CI=4.25 to 7.60). Also, increasing number of previous facilities visited decreased inpatient satisfaction level significantly. For example, satisfaction levels decreased for those who visited only 1, 2 and 3+ facilities prior to KBTH by 0.59% (95% CI= -0.1.1 to 0.17), 0.18% (95% CI= -1.34 to -0.48) and 0.82% (95% CI=-1.36 to - 0.28) respectively (Table 5). Outpatient satisfaction level was significantly influenced by place of residence. Having an urban place of residence significantly decreased outpatient satisfaction by over 10 times compared to satisfaction rates of those residing in a rural area (aβ= - 10.47; 95% CI= -15.29 to -5.66) (Table 5).

**Table 5:** Factors associated with patients' level of satisfaction with the services of doctors and nurses using the Heckman selection model

Variable	Inpatient; 1=Yes, 0=No (selection)	Inpatient Satisfaction raw scores	Outpatient; 1=Yes, 0=No (selection)	Outpatient Satisfaction raw scores
	aβ[95%C%]	aβ[95%C%]	aβ[95%C%]	aβ[95%C%]
**Age**	-0.01[-0.02 to 0.00]	0.01[0.00 to 0.02]*	-0.01[-0.02 to 0.01]	-0.03[-0.13 to 0.08]
**Religion**				
**Islam**	1	1	1	1
**No religion**	0.39[-0.56 to 1.33]	0.84[-0.34 to 3.02]	0.68[-0.51 to 1.87]	3.00[-4.11 to 10.11]
**Christianity**	0.12[-0.24 to 0.48]	-0.15[-0.54 to 0.25]	0.17[-0.24 to 0.58]	-2.07[-6.38 to 2.24]
**Education**				
**Tertiary**	1	1	1	1
**No formal education**	-0.30[-0.75 to 0.16]	0.36[-0.10 to 0.82]	0.33[-0.17 to 0.83]	-2.13[-6.24 to 1.99]
**Primary**	-0.54[-0.97 to -0.10]**	-0.11[-0.56 to 0.34]	-0.15[-0.63 to 0.32]	-1.42[-5.72 to 2.89]
**Middle/ Junior High School**	-0.08[-0.40 to 0.25]	0.001[-0.33 to 0.32]	0.07[-0.28 to 0.42]	-0.81[-3.71 to 2.09]
**Secondary**	-0.16[-0.47 to 0.15]	0.04[-0.25 to 0.34]	0.08[-0.24 to 0.40]	-0.76[-3.49 to 1.97]
**Status**				
**Married**	1	1	1	1
**Never married**	-0.23[-0.59 to 0.13]	0.25[-0.09 to 0.59]	0.15[-0.21 to 0.51]	-3.37[-6.89 to 0.16]
**Co-habiting**	-1.01[-2.67 to 0.66]	5.93[4.25 to 7.60]***	0.60[-0.66 to 1.85]	-4.02[-11.61 to 3.57]
**Divorced/Separated**	-0.10[-0.39 to 0.20]	0.20[-0.11 to 0.51]	0.40[0.06 to 0.74]*	-1.67[-4.38 to 1.05]
**Widowed**	0.11[-0.19 to 0.41]	0.27[-0.06 to 0.60]	0.63[0.26 to 0.99]***	0.80[-2.09 to 3.68]
**Residence**				
**Rural**	1	1	1	1
**Urban**	-0.23[-1.12 to 0.67]	0.52[-0.14 to 1.18]	0.33[-0.33 to 0.99]	-10.47[-15.29 to - 5.66]***
**Currently working**				
**No**	1	1	1	1
**Yes**	0.07[-0.21 to 0.34]	0.07[-0.22 to 0.35]	0.07[-0.23 to 0.36]	0.89[-1.69 to 3.46]
**Income (GHS)**				
**<500**	1	1	1	1
**500-1000**	-0.13[-0.38 to 0.12]	-0.02[-0.29 to 0.25]	0.12[-0.51 to 0.27]	-0.26[-0.78 to 0.27]
**1001-2000**	-0.32[-0.69 to 0.06]	-0.23[-0.59 to 0.13]	-0.38[-0.75 to -0.01]*	-0.05[-0.56 to 0.46]
**2001-5000**	-0.31[-0.67 to 0.05]	-0.00[-0.38 to 0.38]	-0.22[-0.65 to 0.22]	-0.43[-0.96 to 0.10]
**>5000**	-0.30[-0.76 to 0.16]	0.29[-0.22 to 0.79]	-0.56[-0.98 to -0.15]*	-0.33[-0.84 to 0.19]
**First point of call**				
**Health Facility**	1	1	1	1
**Other**	-0.21[-0.57 to 0.16]	0.24[-0.13 to 0.60]	0.42[-0.02 to 0.86]	-0.11[-3.22 to 3.00]
**Previous number of health facilities visited**				
**None**	1	1	1	1
**1 Facility**	-0.05[-0.43 to 0.34]	-0.59[-1.01 to -0.17]**	-0.64[-1.13 to -0.15]**	-2.80[-5.81 to 0.21]
**2 Facilities**	0.19[-0.33 to 0.70]	-0.19[-1.34 to -0.48]***	-1.01[-1.51 to -0.50]***	-1.00[-4.61 to 2.60]
**3+ Facilities**	0.06[-0.52 to 0.65]	-0.82[-1.36 to -0.28]**	-0.96[-1.55 to -0.38]***	-1.56[-6.51 to 3.38]

Hospital visit influenced by another				
**No**	1	1	1	1
**Yes**	0.21[-0.10 to 0.51]	-0.09[-0.40 to 0.22]		
Hospital visit based on personal conviction			0.00[-0.33 to 0.34]	1.24[-1.39 to 3.87]
**No**	1	1	1	1
**Yes**	**0.54[0.18 to 0.89]** **	**-0.33[-0.62 to -0.04]** *	**-0.09[-0.40 to 0.22]**	**0.78[-1.80 to 3.35]**

NOTE: Abbreviation: CI=Confidence Interval; GHS=Ghana Cedis; aα= adjusted coefficient estimate from Heckman Selection model; 1= reference category used for inference. P-value notation:

* p<0.05

** p<0.01

*** p<0.001

## Discussion

The Surgical Department and the National Centre for Radiotherapy, Oncology and Nuclear Medicine of KBTH have not previously surveyed patients with breast cancer on their level of satisfaction with care. The results of this study offer insight into patient levels of satisfaction regarding inpatient and outpatient treatments for breast cancer at KBTH. Breast cancer patients with inpatient and outpatient experience were 61.63% and 74.68%, respectively. These rates are similar to those found in other investigations carried out in related environments. The study notes that differences in inpatient attendance were significantly related to several factors including marital status, number of health facilities previously visited prior to KBTH, and selection of KBTH based on personal conviction, while differences in outpatient attendance were significantly related to marital status, first point of call when their condition started, and the number of health facilities previously visited prior to KBTH.

The measured level of satisfaction was lower compared to the self-reported level for both inpatients (74/100 versus 4.22/5) and outpatients (49.99/100 versus 4.11/5). Plausible explanations could include the fact that the measured levels took into account every aspect of care including waiting times and the state of the lavatories among others while patients may just focus on the nature of their interaction with the service provider when reporting their level of satisfaction.

The measured level of satisfaction for inpatient care was higher (approximately 74%) compared to outpatient (approximately 50%) care. This observed difference was however not statistically significant. The patient satisfaction rate in our study was similar to the 69.5% patient satisfaction with healthcare delivery in Accra, Ghana reported by Odokor *et al.*^15^

An American study assessing satisfaction with breast cancer follow-up care provided by family physicians reported that 73.4% of respondents were “extremely satisfied” with their care.^16^ A Tanzanian study looking at patient satisfaction rates regarding outpatient healthcare services reported 20% of outpatients to be satisfied with the level of care received.^17^ It can be challenging to apply the findings of previous studies investigating patient satisfaction to the findings noted in this study given differences in patient population, disease process, country of study, and methods used to estimate healthcare service satisfaction.

Identifying variables that impact patient attendance and patient satisfaction scores is critical to making healthcare delivery more patient-centered and improving quality of care. This enables healthcare service providers to tailor interventions to fit the unique needs of various patient groups levels.^16^ After controlling for selection bias, age, marital status, number of previous health facilities visited, and hospital selection based on personal conviction were found to be significant predictors of inpatient satisfaction among breast cancer patients at KBTH. Increasing age, cohabitation, and selection of hospital based on personal conviction were linked to higher hospital satisfaction, whereas increasing income and increased number of previous facilities visited was linked to lower inpatient satisfaction. These results are in line with those of earlier research that identified similar comparable factors influencing patient satisfaction.^18^,^19^ El Marnissi *et al.*^11^ found that individual characteristics such as age, education, occupation, and marital status influence patients' level of satisfaction^20^, while both Thind *et al.* and Batbaatar *et al.* found age to be the most important and consistent predictor of patient satisfaction.^21^, ^16^

This study also revealed that the place of patient residence was a significant predictor of outpatient satisfaction, with lower levels of satisfaction reported by urban residents. This result is in line with earlier research also describing a connection between patient satisfaction and patient residence.^18^,^19^ Urban dwellers are generally accustomed to better amenities than patients from more rural locations and may thus have relatively higher expectations that could partly account for their lower patient satisfaction levels.

## Conclusion

The study offers insightful information about attendance and levels of both measured and self-reported satisfaction among breast cancer patients at KBTH in the inpatient and outpatient setting, as well as the factors influencing both attendance and satisfaction. Addressing the variables that affect attendance and satisfaction levels is key to enhancing the provision of healthcare services for any patient population. Given that breast cancer diagnosis and treatment can negatively impact the psycho-social well-being of patients, understanding and improving patient satisfaction among breast cancer patients is a way that providers can bolster the emotional wellbeing of patients. Improvement in patient satisfaction at KBTH among outpatients, specifically addressing concerns of more urban patients, is an area for future improvement to enhance healthcare delivery.
